# Preference test of sound among multiple alternatives in rats

**DOI:** 10.1371/journal.pone.0197361

**Published:** 2018-06-13

**Authors:** Ryo Soga, Tomoyo Isoguchi Shiramatsu, Hirokazu Takahashi

**Affiliations:** Research Center for Advanced Science and Technology, The University of Tokyo, Tokyo, Japan; Tokai University, JAPAN

## Abstract

Conditioned place preference (CPP) tests in rodents have been well established to measure preference induced by secondary reinforcing properties, but conventional assays are not sensitive enough to measure innate, weak preference, or the primary reinforcing property of a conditioned stimulus. We designed a novel CPP assay with better sensitivity and efficiency in quantifying and ranking preference of particular sounds among multiple alternatives. Each test tone was presented according to the location of free-moving rats in the arena, where assignment of location to each tone changed in every 20-s session. We demonstrated that our assay was able to rank tone preference among 4 alternatives within 12.5 min (125 s (habituation) + 25 s/sessions × 25 sessions). In order to measure and rank sound preference, we attempted to use sojourn times with each test sound ($\bar{T}$), and a preference index (PI) based on transition matrices of initial and end sounds in every session. Both $\bar{T}$ and PI revealed similar trends of innate preference in which rats preferred test conditions in the following order: silence, 40-, 20-, then 10-kHz tones. Further, rats exhibited a change in preference after an classical conditioning of the 20-kHz tone with a rewarding microstimulation of the dopaminergic system. We also demonstrated that PI was a more robust and sensitive indicator than $\bar{T}$ when the locomotion activity level of rats became low due to habituation to the assay repeated over sessions. Thus, our assay offers a novel method of evaluating auditory preference that is superior to conventional CPP assays, offering promising prospects in the field of sensory neuroscience.

## Introduction

Preferences are shaped both by innate characteristics of the sensory system and by personal experience throughout life and, in turn, may affect neural activities in the brain. Therefore, gauging preference in animal studies offers valuable insights into neural representation.

For visual stimuli, alternative forced choice tasks have proven to be valuable tools for determining preference in animals. For example, monkeys have exhibited preference for complex visual stimuli [1–3], symmetrical and regular patterns [4], movies rather than still images [5], and social contents, such as conspecific faces [2, 6, 7], female perinea, and faces of high-status monkeys [8]. Additionally, rodents have exhibited visual preference for novel objects [9, 10] and movies of conspecific social behaviors [11]. Further, alternative force choice tasks have been used to show that preference for a particular style of painting can be induced in pigeons [12–15], and surprisingly, that mice [16] and songbirds [17] may have certain innate visual preferences. However, to measure preference in these alternative forced choice tasks requires that alternatives appear simultaneously, which makes this methodology difficult to apply to non-visual fields.

Conditioned place preference (CPP) is another well-established test that, since the discovery of rewarding effects of brain stimulation [18], has been often used to characterize reinforcement effects, specifically for drugs of abuse [19–21]. In the CPP test, one compartment in a test arena is paired with a primary reinforcer and, following conditioning, the secondary reinforcing properties become associated with the compartment due to Pavlovian contingency; consequently, sojourn time within the compartment becomes a measure of this reinforcing. The CPP test is often modified to measure preference for a conditioned context. For example, odor served as an effective contextual conditioned stimulus in rats [22], as has music in goldfish [23], songbirds [24], pigeons [25], rats [26–29], and monkeys [30]. Thus, these assays have proven valuable methods for measuring preference as induced by secondary reinforcing properties. However, traditional CPP tests are not sensitive enough to measure innate, weak preferences or to measure preference for the primary reinforcing property of a conditioned stimulus.

Current applications of CPP tests have several problems that if addressed, would improve these assays. First, because differences in sojourn time across the two compartments are usually quantified as a measure of preference in a pairwise manner, CPP is too time-consuming to rank preferences of multiple alternatives. Second, locomotion activity has a profound effect in these assays [19]. Specifically, locomotion is often much more costly for the test subjects than the value of the reinforcing properties of contextual stimulus. Further, during the test, locomotion activity level decreases with time [31], and a strong initial bias of preference is sometimes observed, which can complicate interpretation of reinforcing effects of the conditioned stimulus [21, 32].

To address these limitations of current application of CPP, we designed a novel CPP assay that would allow quantification and preference ranking of multiple alternatives of test tones with better sensitivity and efficiency. In this novel assay, rats were allowed free movement throughout the arena where each tone was presented according to specific location, but where the assignment of location to each tone changed with every 20-s session. We hypothesized that rats would stay longer in locations with preferred tones, would seek preferred tones, and would move away from uncomfortable tones. In addition to sojourn times for each test tone, we derived a preference index (PI) based on transition matrices of initial and end tones in every session. We hypothesized that locomotion activity level of test rats would have less influence over PI than over sojourn time because the index places a greater emphasis on self-active sessions than on motionless sessions. Using our novel assay, we first sought to confirm that in our setup rats still indicated their well-documented preference for silence over meaningless, unfamiliar sounds [28, 33, 34]. Next, we examined whether our assay was sufficiently sensitive to reveal preference among rats for particular test tones. We then demonstrated that our assay could detect modifications of this preference when a particular tone was paired with rewarding microstimulation of the dopaminergic system in the midbrain [35–37].

## Material and methods

This study was conducted in strict accordance with ‘‘Guiding Principles for the Care and Use of Animals in the Field of Physiological Science” published by the Japanese Physiological Society. The experimental protocol was approved by the Committee on the Ethics of Animal Experiments at the Research Center for Advanced Science and Technology, the University of Tokyo (Permit Number: RAC130107). All surgery was performed under isoflurane anesthesia, and all efforts were made to minimize suffering. During experiments, each animal was individually housed in a polycarbonate cage (276×445×204 mm^3^) (CL-0108-1; CLEA Japan, Inc., Tokyo, Japan) with 4-cm-thick nesting material. The breading area was constantly kept at 21°C and under a 12:12 hours day-night cycle. Dry food and water was provided ad libitum. At the termination of experiment, animals were euthanized with an overdose of pentobarbital sodium (160 mg/kg, i.p.).

### Design of experimental setup

**Fig 1:**
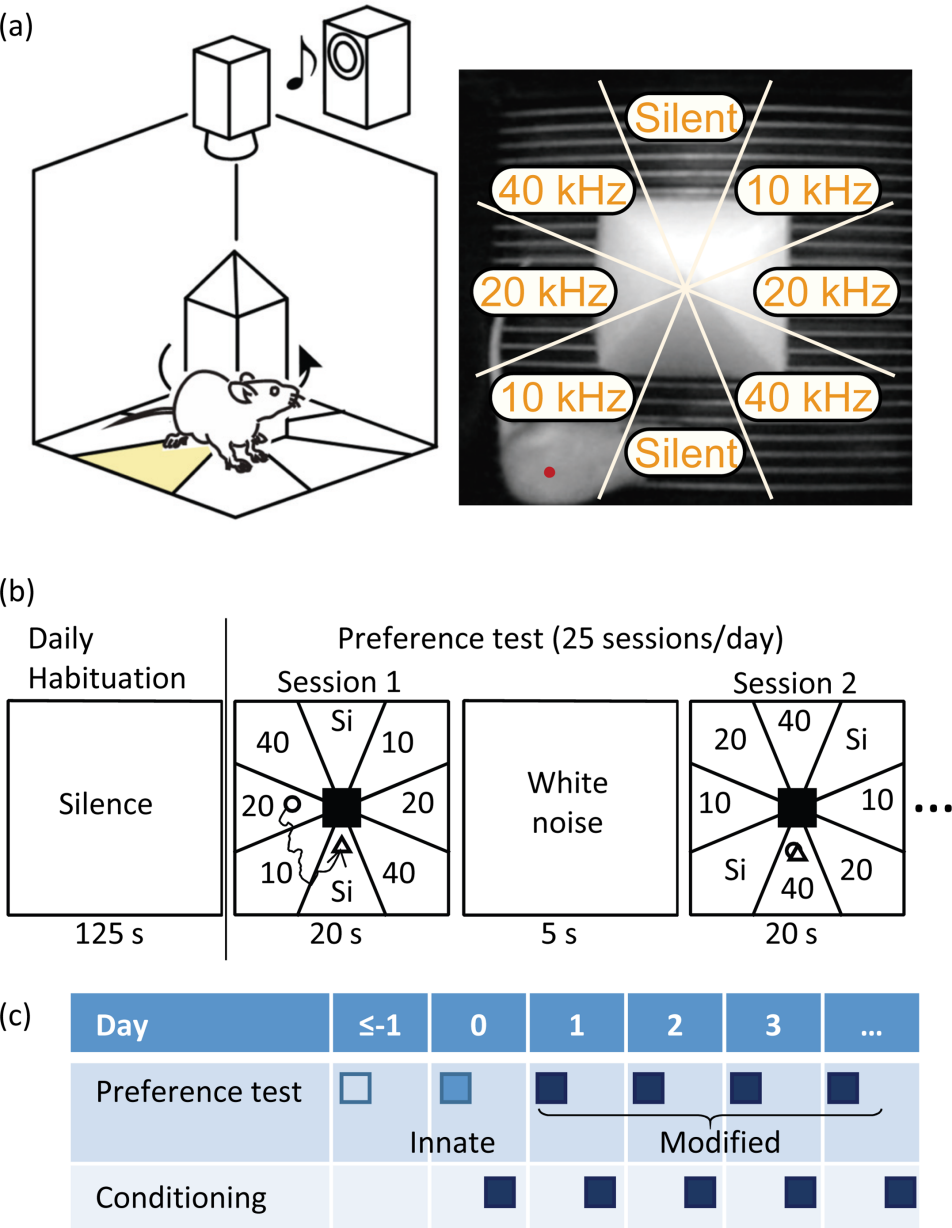
Experimental setup and design. (a) Schema of setup. A camera tracked the position of a test rat in the arena, where a square pillar stood at the center. The arena was virtually divided into eight subfields, to each of which a different auditory environment was assigned. One of these tones was presented depending on the location of the gravity center of a test rat shown by a red dot. (b) Daily protocol. A habituation period was given first. A single session of sound preference test lasted 20 s, and 25 trials were repeated daily. Each session had a different spatial assignment of tone frequency. Each session was interleaved by a 5-s white noise period. (c) Schedule of the experiments. Preference was measured daily. Day 0 was defined as the first day of conditioning, which associated a 20-kHz tone with rewarding microstimulation of the dopaminergic system. Innate preference was characterized on day 0. A preference test on day 1 or later examined whether and how the conditioning modified the innate preference.

Fig 1A depicts the schema of our experimental setup. Behavioral experiments were conducted using a custom-made chamber (35×35×35 cm^3^) (OPFZ-3001; O’hara & Co. Ltd., Tokyo, Japan) placed in a soundproof booth (80×70×70 cm^3^) (Japan Shield Enclosure Co. Ltd., Osaka, Japan). A camera on the chamber ceiling tracked the position of test rat and captured images every 60 ms. These images were binarized to identify the gravity center of the silhouette, which defined the position of the rat. A square pillar (7×7×25 cm^3^) was placed at the center of the arena and the rat moved freely around the pillar. A speaker mounted above the chamber (Technics EAS-10TH800; Matsushita Electric Industrial Co. Ltd., Osaka, Japan) provided sound stimuli. Prior to the experiments, acoustic calibration was performed with a 1/4-inch microphone (4939; Brüel & Kjaer, Nærum, Denmark) at the center of the arena at a height of 3 cm (i.e., head height of rats). We then measured sound intensity at multiple locations in the arena and confirmed that the difference of intensity was less than 3 dB.

In daily tests, rats were first habituated to the arena for 125–625 s without any sound presentation, and 25 sessions of preference testing were carried out. The arena around the pillar was virtually divided into 8 subfields and, based on the specific subfield in which a test rat was located, one of the following acoustic environments was given: continuous tones at 60 dB sound pressure level (SPL) of (i) 10 kHz; (ii) 20 kHz; (iii) 40 kHz; or (iv) silence (Fig 1A). The allocation of particular acoustic environments to each subfield changed randomly every session, with the restriction that the tone at the beginning of the session be different from the tone at the end of last session (Fig 1B). A single session was 20 s long and was interleaved with while noise at 30 dB SPL for 5 s.

### Indices of preference

To quantify preference in rats for particular auditory environment, normalized sojourn time ($\bar{T}$) and preference index (PI) were defined. Similar to previous studies utilizing conventional CPP assays [19–21], the use of $\bar{T}$ as an indicator of preference is based on the hypothesis that rats would stay longer in subfields presenting the preferred auditory environment of the animal. However, because the rats often sat still and did not move at all during a session, the initial condition of the rat might profoundly bias sojourn times. To avoid this bias, $\bar{T}$ was derived for each condition such that a time rate spent in each condition was normalized with the probability of initial condition: i.e.,

$$\bar{T}(X)=\frac{\mathrm{Pr}\left(X\right)}{\mathrm{Pr}\left(S=X\right)}$$

where X denotes the condition of auditory environment, and S denotes the initial condition in each session. To derive the time rate spent in each condition, i.e., Pr(X), the sojourn time of X during test was divided by the total time, i.e., 500 s for 25 sessions. An initial condition in each session was chosen with care to equal the probability of each initial condition at the end of daily test, i.e., Pr(S = X) = 1/4. Yet, this was not always possible because of the restriction that each initial condition be different from the end condition of the last session. To compensate this bias in the initial condition, Pr(X) was finally normalized by Pr(S = X).

However, even with this normalization, $\bar{T}$ could not evaluate preference adequately when rats sat still in most sessions. Therefore, PI was designed to put more weight on self-active sessions. Rats might actively seek out and find a subfield with their preferred auditory environment, as opposed to moving away from a subfield with their non-preferred environment. To evaluate this tendency, PI was defined as

$$PI(X)=\mathrm{Pr}\left(G=X|S=\bar{X}\right)-Pr(G=\bar{X}|S=X)$$

where X is the condition of auditory environment, and S and G are the initial and end auditory conditions in each session, respectively, and $\bar{X}$ is the complementary condition of X (e.g., when X is 10 kHz, $\bar{X}$ consists of silence, 20 kHz and 40 kHz). $\mathrm{Pr}\left(G=X|S=\bar{X}\right)$ and $Pr(G=\bar{X}|S=X)$ were the inflow and outflow probability of X, respectively, which were obtained daily at the end of all sessions, i.e., 25 sessions. PI(X) = 1 when rats always ended sessions within the subfield presenting X, i.e., G = X, while PI(X) = -1 when G = $\bar{X}$ in all sessions.

### Innate preference test

Using the experimental setup and indices defined above, we tested 41 naïve male Wistar rats (Tokyo Laboratory Animals Science Co., Ltd., Tokyo, Japan), at postnatal week 9 or 10 and with a body weight of 250 to 350 g, to examine preference of naïve rats.

### Modification of preference through classical conditioning

Classical condition was done by pairing a 20-kHz tone with stimulation of the ventral tegmental area (VTA). In 31 of the rats used in our innate preference testing, a custom-made bipolar electrode was implanted in the VTA to electrically activate the dopaminergic system as a reward.

For implantation, rats were anesthetized with isoflurane (3.5% at induction and 3% for maintenance) and were fixed in a stereotaxic frame (SR-50; Narishige Group, Tokyo, Japan) using ear bars. Atropine sulfate (0.1mg/kg) was administered at the beginning of the surgery to reduce the viscosity of bronchial secretions. A skin incision was made at the beginning of the surgery under local anesthesia of xylocaine (0.5 ml), and the parietal cranium was surgically exposed. The cranium and dura over the target site were removed, and 3 small craniotomies were conducted to implant anchoring screws. An electrode bundle was inserted into the right VTA (-5.2 mm anterior-posterior (AP) and 0.95 mm medio-lateral (ML) from bregma at a depth of 8.5 mm from the surface of brain). The electrode bundle was composed of a pair of Teflon-insulated stainless wires with the bare diameter of 110 μm (Nilaco Corp., Tokyo, Japan). The wires were inserted into a 26 gauge injection needle serving as a guide cannula. The cannula and anchoring screws were fixed together on the skull with dental cement (Unifast Trad; GC Corp., Tokyo, Japan).

A post-operative recovery period was given for 3 days or more. Once animals had recovered from surgery, self-stimulation behaviors were quantified for 3 min to confirm effectiveness of VTA stimulation. The self-stimulation experiments were carried out in an operant chamber (12×25×35 cm^3^) (O’hara & Co. Ltd.), which had a 2.5-cm diameter nose-poke (NP) hole on the wall. Each NP triggered VTA stimulation, which was a 200-ms, 100-Hz train of charge-balanced biphasic pulses each with a current of 0.4 mA and duration of 0.2 ms. Rats exhibiting more than 75 nose poking during 3-min period of testing were included in the classical conditioning group. Consequently, the rats with the top 10 highest NP frequencies were used as the classical conditioning group.

For classical conditioning, we paired a 20-kHz tone with VTA stimulation, and examined whether preference was modified through conditioning. The conditioning was conducted in another experiment chamber (35 × 35 × 35 cm^3^) (O’hara & Co. Ltd.). In the conditioning, a 20-kHz, 60-dB SPL tone was presented for 30 s as a conditioned stimulus (CS), and the last 200 ms of the CS was presented with the unconditioned stimulus (US), VTA stimulation, to create an association between the two stimuli. The parameters of VTA stimulation were identical to those delivered during self-stimulation. These CS-US pairs were presented 200 times daily during the conditioning with an inter-stimulus interval between each CS-US pairing of 31–35 s. The total duration of a conditioning session was approximately 100 min.

For the first 2 days or more, innate preference was characterized. Thereafter, the classical conditioning above was conducted, followed by a daily preference test; hence, the effect of conditioning would appear in the preference test on the next day. We defined day 0 as the first day of conditioning, when innate preference was characterized; thereafter (i.e., day 1 or later), we quantified whether and how the conditioning modified preference (Fig 1C).

## Results

**Fig 2:**
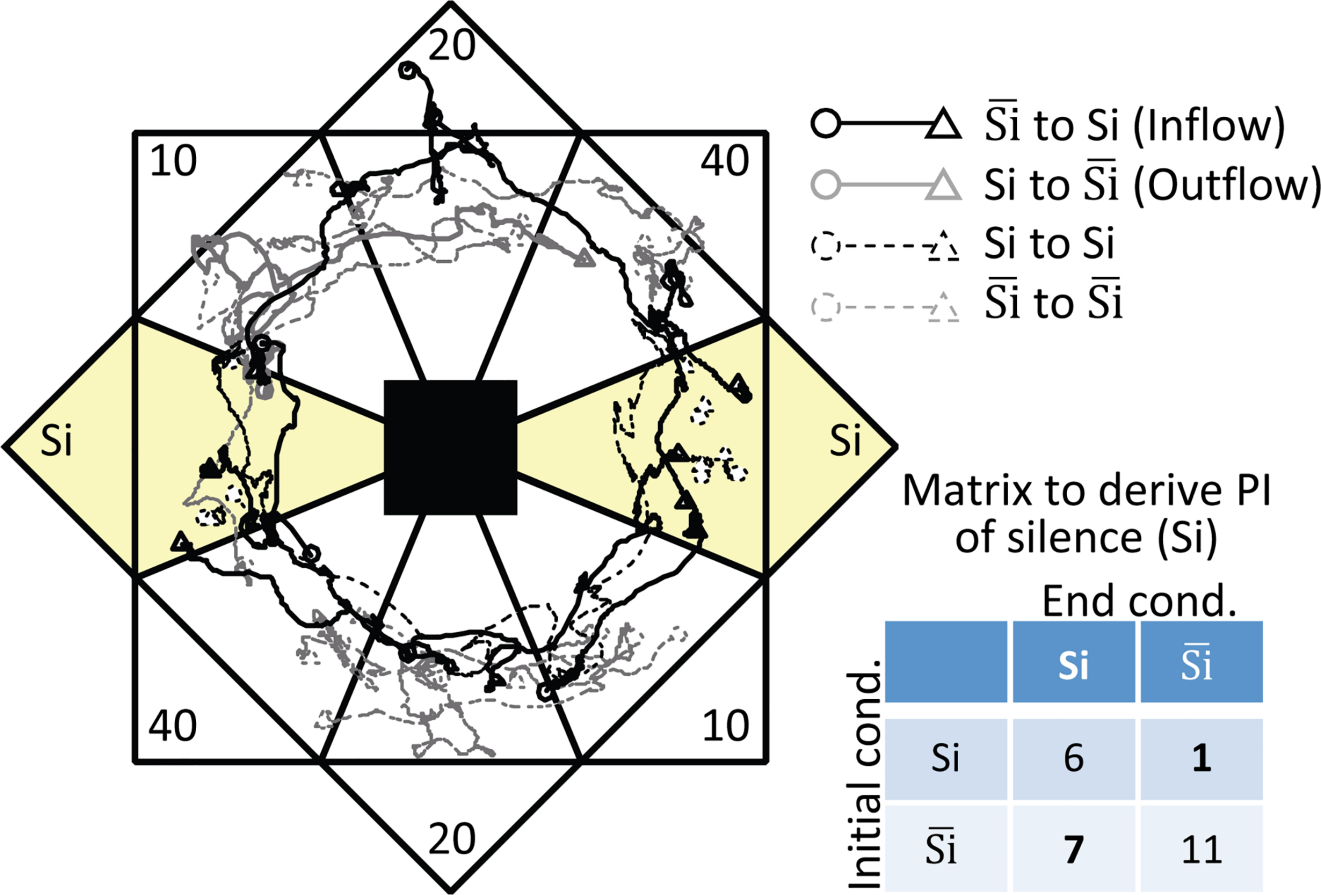
Representative data. Representative paths of 25 sessions on a given day. Circles and triangles indicate initial and end positions, respectively, in a given session. Solid and broken paths indicate self-active and motionless sessions, respectively. To derive the preference index (PI) of silence (Si), a transition matrix of initial and end conditions was used, as shown in the lower right section of the figure. $\bar{Si}$ consisted of 10-, 20-, and 40-kHz tones. From self-active sessions (i.e., the non-diagonal components), the frequency of inflow (black) and outflow (gray) was quantified, and the difference between the inflow and outflow rate was defined as the PI of silence; in this example, PI = 7/18-1/7 = 0.25.

Fig 2A depicts representative paths from 25 sessions on a given day in the innate preference test, from which the behavioral indices, $\bar{T}$ and PI, were derived. From this representative data, a matrix of the initial and end conditions from each session was examined to derive PI. In this example, a PI of silence was derived as 7/18–1/7 = 0.25. A positive PI indicates the inflow of the test animals to a given condition during the session was more frequent than their outflow. Diagonal elements in the matrix were ignored in PI derivation, because most of these elements corresponded to motionless sessions.

**Fig 3:**
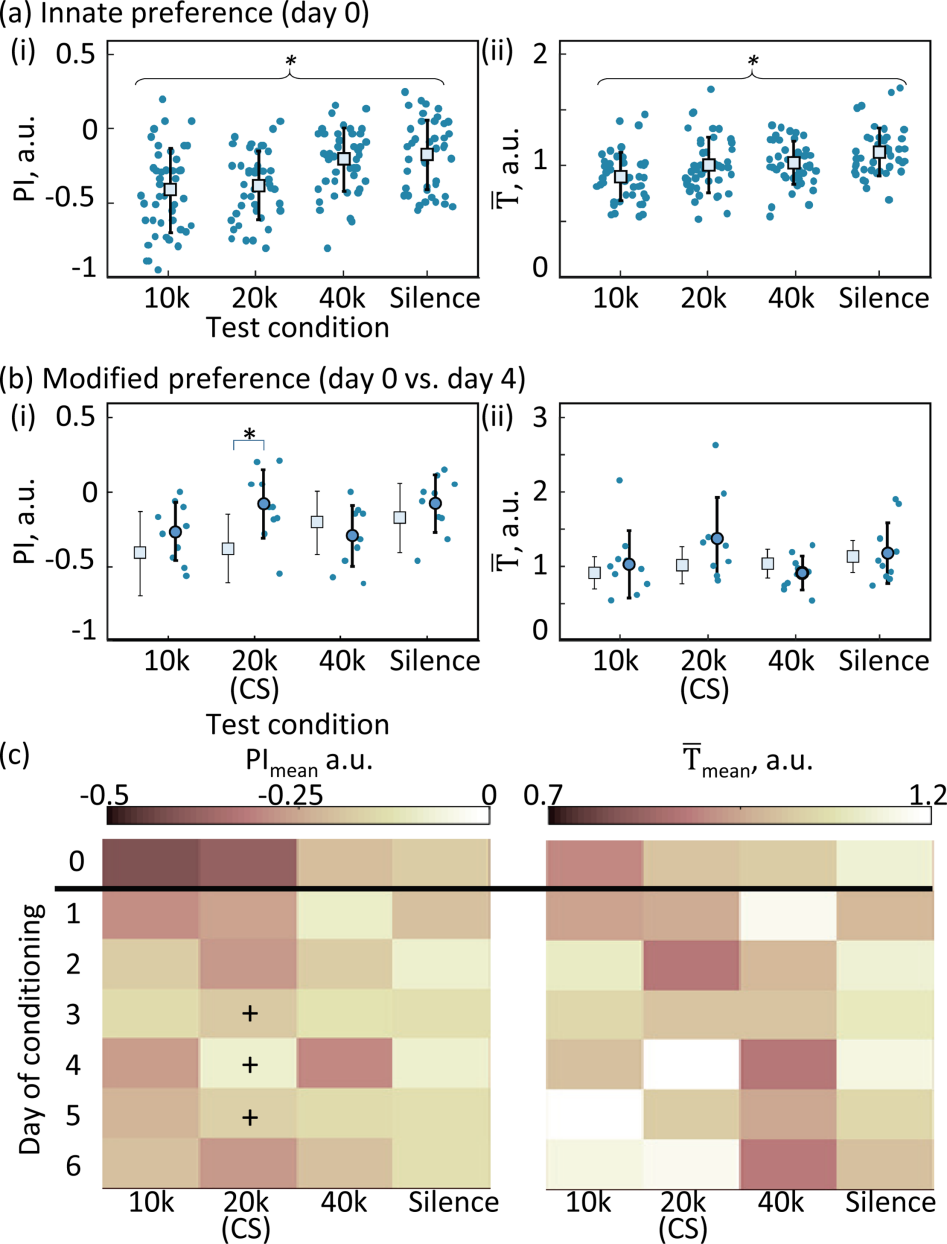
Innate and modified preference of rats for specific tones. (a) Innate preference. (i) Preference index (PI) measurement. (ii) Normalized sojourn time ($\bar{T}$) measurement. The means and standard errors are given. Dots indicate data from individual subjects. Asterisks indicate statistical significance among test conditions (one-way ANOVA). (b) Modified preference. PI and $\bar{T}$ were measured after 4 days of classical conditioning, where a 20-kHz tone was paired with rewarding microstimulation to the ventral tegmental area (VTA). For comparison, square symbols indicate innate preference adapted from (a). (c) Daily data of preference test. Conditioning induced a significant difference in PI, but not in $\bar{T}$ (two-way ANOVA). Plus marks indicate significant increase of preference for the 20-kHz tone on a given day as compared to day 0 (post-hoc t-test with Bonferroni correction).

In the innate preference test (Fig 3A), significant differences between the conditions were observed both in PI (Lilliefors test for normality, p > 0.05; one-way ANOVA, F_3, 172_ = 11.05, p = 1.1e-6) and in $\bar{T}$ (Lilliefors test for normality, p > 0.05; one-way ANOVA, F_3, 172_ = 7.32, p = 1.2e-4). Consistent with previous studies using classical CPP assays, an innate preference was observed for silence in PI (post-hoc t-test with Bonferroni correction: silence vs. 10 k, p = 0.00083; silence vs. 20 k, p = 0.0013) and in $\bar{T}$ (silence vs. 10 k, p = 0.0028); furthermore, PI suggested preference difference among tone frequencies, 40-kHz tone being preferred to 10-kHz and 20-kHz tones (40 kHz vs. 10 kHz, p = 0.00032; 40 kHz vs. 20 kHz, p = 0.0019).

We next attempted to modify this innate preference through classical conditioning. NP frequency in the self-stimulation experiment was examined to confirm how effectively VTA stimulation served as a reward. In 31 test rats, the frequency of NP over 3 min ranged widely from 0 to 350 (median, 68; interquartile range, 33–147). Among them, 14 rats that made NP more than 75 times over 3 min were included in the classical conditioning group, in which a 20-kHz tone was paired with VTA stimulation to modify the innate preference in these animals. In this conditioning group, 4 rats were excluded from the following analyses because stimulation electrodes were damaged during experiments, and thus 10 rats were eventually investigated.

Fig 3B shows scatter plots of PI and $\bar{T}$ on day 4, suggesting that preference of 20-kHz tone selectively increased as compared to that in the innate preference test. For statistical tests, two-way ANOVA quantified effects in the auditory condition and conditioning day. For PI, significant main effects were observed in both the auditory condition (F_3, 388_ = 5.39, p = 0.0138; post-hoc power analysis, 1- β = 0.259) and the conditioning day (F_6, 388_ = 4.20, p = 0.0004; 1- β = 0.429) (Fig 3C). Further, PIs of 20 kHz on days 3–5 were significantly higher than the innate PI of 20 kHz (post-hoc t-tests with Bonferroni correction: day 3, p = 0.0486; day 4, p = 0.0015; day 5, p = 0.027; scatter plots on day 4 in Fig 3B). In contrast, no significant main effects were observed for $\bar{T}$ of either the auditory conditions (F_3, 388_ = 0.107, p = 0.107; 1- β = 0.119) and day (F_3, 388_ = 0.03, p = 0.99; 1- β = 0.0829). Thus, PI appeared to be more sensitive than $\bar{T}$ as a measure of preference.

**Fig 4:**
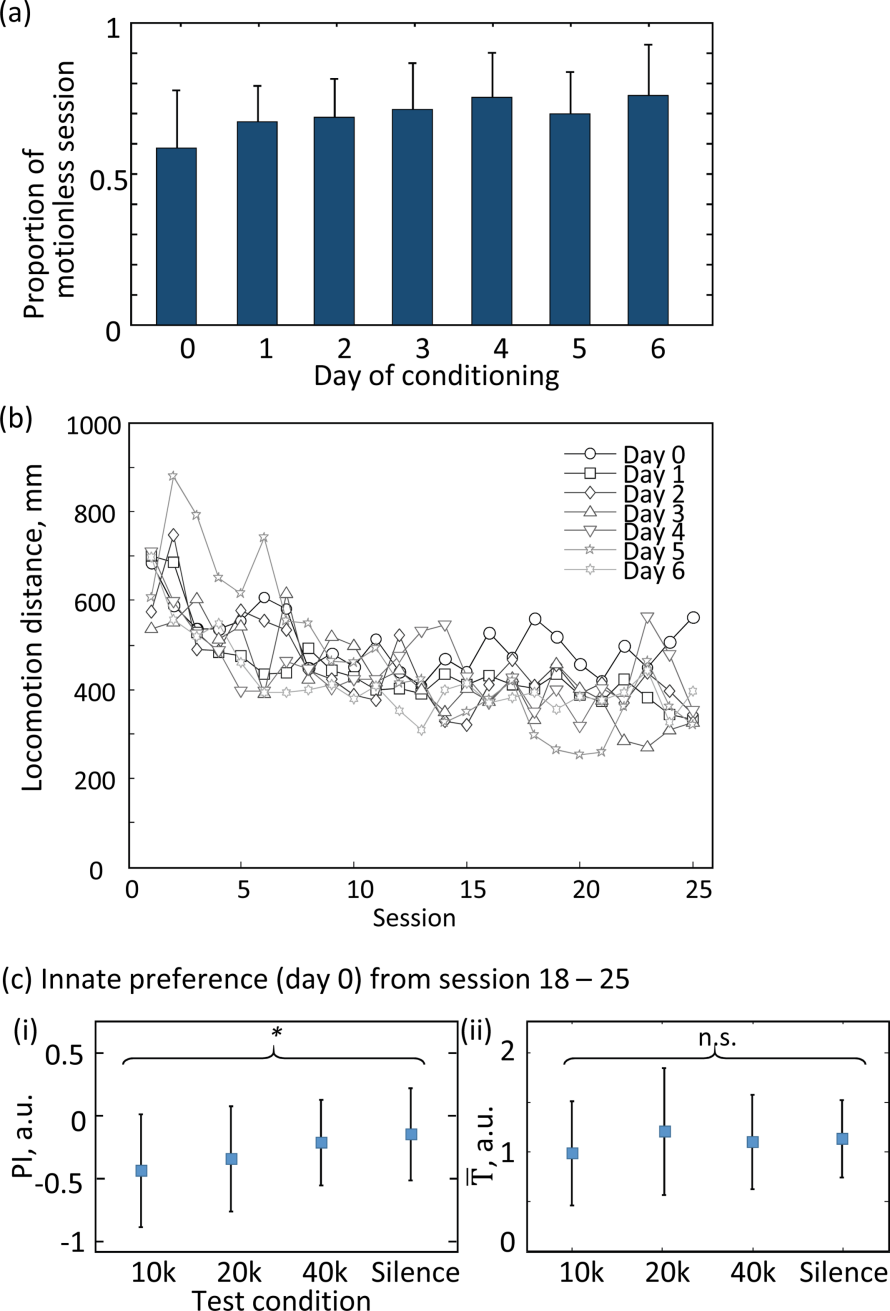
Effects of locomotion activity on preference test. (a) Proportion of the session in which the rats was motionless. The means and standard deviations are given. (b) Path length of locomotion. The mean across subjects in a given session on a given day is shown. Locomotion activity decreased over sessions within a given day, but not over days of conditioning (two-way ANOVA). (c) Innate preference derived from the last 1/3 of the sessions (18–25), in which locomotion activity was low. Innate preference was still uncovered in PI (i), but not in $\bar{T}$ (ii) (one-way ANOVA).

The poor power of $\bar{T}$ in the preference test was likely due to low locomotion activity with repetition of the tests. In the preference tests, rats did not move beyond a given subfield (S = G) in approximately 70% of sessions (Fig 4A). The length of the locomotion path decreased over the sessions in a given day, but did not change across the days (Fig 4B) (two-way ANOVA: F_24, 2424_ = 6.99, p < 10^−6^ for session; F_7, 2424_ = 0.81, p = 0.581 for day). We reevaluated innate preference from the last 1/3 of the sessions (18–25) on day 0, where the total length of locomotion (mean ± s.d. = 529 ± 249 mm) was significantly shorter than that of the first 1/3 of the sessions (384 ± 202 mm) (t-test, p = 3.2e-6). We found that PI still varied across the auditory conditions (one-way ANOVA, F_3, 175_ = 4.7, p = 0.0035; 1- β = 0.906), while $\bar{T}$ showed no significant difference (F_3, 175_ = 1.37, p = 0.25; 1- β = 0.376). These data support our hypothesis that preference can be better measured from self-active sessions, and that PI is therefore better than $\bar{T}$ to measure preference in our CPP assays.

## Discussion

We designed a novel CPP assay to quantify and rank preference of auditory stimuli among multiple alternatives (Fig 1). Each test tone was presented according to the location of free-moving rats in the arena, where the assignment of location changed for each tone every 20-s session. Preference was quantified by sojourn times ($\bar{T}$) within each test tone location and by preference index (PI) based on transition matrices of initial and end tones in every session (Fig 2). Both $\bar{T}$ and PI revealed a similar trend of innate preference, showing that rats preferred auditory conditions in the following order: silence, 40-, 20-, and 10-kHz tones. Further, both $\bar{T}$ and PI detected a change in preference after the classical conditioning in which a 20-kHz tone was paired with VTA microstimulation (Fig 3). PI was a more robust and sensitive indicator of preference than $\bar{T}$ under conditions when the locomotion activity level of the rats became low due to habituation to the assay repeated over sessions (Fig 4). These results demonstrated the ability of our assay to evaluate auditory preference more efficiently than a conventional CPP assay.

Consistent with results from previous studies, our data showed that rats preferred silence to meaningless, unfamiliar sounds [28, 33, 34] (Fig 3A). Furthermore, according to our data, rats exhibited innate preference among different tones, with rats preferring the 40-kHz tone over the 10-kHz tone. For these tones, negative PIs indicate that an outflow from a given tone subfield was more frequently observed than an inflow to the field, suggesting that the resultant ranking quantified the repulsiveness of a tone the rat wished to avoid rather than preference for a tone the rat wished to encounter. This degree of repulsiveness is likely to correspond to perceptual loudness because emotionally negative stimuli are perceived as louder than positive stimuli [38]. Some physiological properties in the auditory system of rats could make 10-kHz tone seem louder than others. Specifically, at the level of the auditory cortex in rats, high-intensity, mid-frequency tones (e.g., 80 dB SPL, 8 kHz) recruited the largest number of neurons [39–42]. This property of recruitment function originates partially in the mechanical characteristics of cochlea [43, 44]. Thus, the innate preference observed in rats during the present experiments supports previous findings that rats prefer a quiet environment.

In our assay, classical conditioning pairing a 20-kHz tone with VTA microstimulation increased preference of conditioned stimulus (Fig 3). Interestingly, preference for the 20-kHz tone evolved gradually and became most obvious on day 4; yet this statistical significance disappeared on day 6. In our assay, because the classical conditioning was conducted in a different chamber than the CPP test, repetitions of the CPP test over days were likely to cause extinction in the CPP apparatus (i.e., rats learned and became aware that no reward was provided) [45].

Our assay has several advantages over conventional CPP assays. First, our assay was able to rank preference among 4 alternatives within 12.5 min (125 s (habituation) + 25 s/sessions × 25 sessions) (Fig 1B). Conventionally, test duration of CPP is typically 10–15 min, in which sojourn times among two compartments are usually quantified as a measure of preference; therefore, to rank 4 alternatives in a pairwise manner would take 60–90 min over multiple days in conventional CPP assays [19]. Second, our apparatus was able to exclude an initial bias, because the relationship between place and conditioned stimuli was randomly assigned and varied every 25 s. Conventional CPP tests commonly used a shuttle box with two highly distinctive compartments (e.g., black vs. white), and thus, a strong initial bias of preference is sometimes observed, which may have profound effects on CPP test results [21, 32]. Third, our PI index was more robust over locomotion activity than sojourn time (Fig 4), because our PI placed more emphasis on self-active sessions rather than motionless sessions. Locomotion activity level commonly decreases during the test as time proceeds [19, 31]. When the cost of locomotion for test subjects becomes more expensive than reinforcing properties of contextual stimulus, sojourn time is not an adequate measure of preference.

Yet, there is a possibility that our assay measures a different aspect from the conventional CPP tests. Subjects in our assay made an active choice among possible tones during tests, and therefore, the behavior under test was essentially conditioned reinforcement. On the other hand, the conventional CPP consisted of pre- and post-tests without stimulus presentation to provide evidence of place or context preference being made. Further experiments is still required to address whether and how our assay is different from conventional CPP tests in preference measurements.

We believe that gauging preference using this novel assay will offer valuable insights in animal studies. For example, consonance is preferred either innately [46] or due to cultural exposure [47], while hard sounds of a sharp object scraping across a slate surface are innately uncomfortable [48]; these preferences may be produced by a particular neural representation in the sensory system, and therefore may be preserved across species [49]. Humans also have long enjoyed music [50–52]. Conventional preference tests have demonstrated that various species are able to discriminate different music as context stimuli [23–30], but the detection power of these tests is still not sufficient to uncover whether and how a specific spatio-temporal structure in particular samples of music has primary reinforcing properties. Furthermore, individual differences of innate preference may emerge depending on early experience. In rodents, biased preference has been shown to develop with early music exposure between postnatal days 15 and 24 [33, 34], but not with exposure to tone pulse patterns [53]. Further, exposure to music in the perinatal period altered signaling of brain-derived neurotrophic factor and enhanced learning performance in mice [54, 55], suggesting that specific spatio-temporal structures of sounds are able to appeal to sensory systems in the brain and develop preference. Thus, the present behavioral assay combined with physiological experiments offers promising prospects for elucidating the underlying neural mechanisms of the development of these previously demonstrated responses to sound.

## Supporting information

S1 Fig Dataset. (XLSX)
